# Supplementary material for: VEFill: accurate and generalizable deep mutational scanning score imputation across protein domains
Source: Mol Syst Biol. 2026 Mar 20;22(6):979–1002. doi: 10.1038/s44320-026-00203-y (PMC13230771; doi:10.1038/s44320-026-00203-y)
Supplement: Supplementary file 6 — Appendix [file 44320_2026_203_MOESM6_ESM.pdf]

Appendix

VEFill: accurate and generalizable deep mutational scanning score imputation across protein domains

Table of contents

Appendix Table S1. List of the 25 amino acid substitution matrices used in VEFill................................ 1-3

Appendix Table S2. Performance comparison of different model architectures using the same input feature set.. 4

Appendix Figure S1. Schema of the custom PostgreSQL database developed for centralized storage and structured access to all model input features..5

Appendix Figure S2. Performance metrics of the general cross-protein model trained and evaluated on various feature sets for DMS score imputation.. 6

Appendix Figure S3. Correlation between average observed DMS scores and average prediction error across amino acid positions.. 7

Appendix Figure S4. VEFill predictive accuracy versus the theoretical noise ceiling for 139 DMS datasets.. 8

Appendix Figure S5. Effect of excluding high-uncertainty DMS variants from the 140-domain dataset on VEFill performance.. 9

Appendix Figure S6. Effect of the number of mutations per position on VEFill performance..................... 9

Appendix Figure S7. Comparison of predictive performance metrics of the general cross-protein model (trained on the Human Domainome 1 dataset) across external stability-based assays and activity-based assays..10

Appendix Figure S8. Comparison of predicted and experimentally measured normalized DMS scores for the general cross-protein model trained on the Human Domainome 1 dataset, tested on external stability-based assays (A–C) and activity-based assays (D–H). 11-12

Appendix Figure S9. Performance of full-feature versus reduced-feature set models trained on 140 and 521 domains, evaluated on Human Domainome 1 and unseen full-length proteins..................................13

Appendix Figure S10. Performance metrics for the zero-shot general cross-protein model trained without positional mean DMS scores, evaluated on external stability-based and activity-based assays................14

Appendix Figure S11. Zero-shot predictions (without positional mean DMS scores) versus experimentally measured normalized DMS scores for external assays.. 15-16

Appendix Table S1. List of the 25 amino acid substitution matrices used in VEFill. Each row includes the matrix name, its source (Biopython or external repository), the corresponding VEFill feature name, and the original citation.

Substitution matrix name	Source	VEFill feature name	Citation
Benner22	Biopython (substitution_matrices .load('BENNER22'))	benner22	Benner SA, Cohen MA, Gonnet GH. Amino acid substitution during functionally constrained divergent evolution of protein sequences. *Protein Engineering*. 1994;7(11):1323–1332. doi:10.1093/protein/7.11.1323. PMID: 7700864.
Benner6	Biopython (substitution_matrices .load('BENNER6'))	benner6	Benner SA, Cohen MA, Gonnet GH. Amino acid substitution during functionally constrained divergent evolution of protein sequences. *Protein Engineering*. 1994;7(11):1323–1332. doi:10.1093/protein/7.11.1323. PMID: 7700864.
Benner74	Biopython (substitution_matrices .load('BENNER74'))	benner74	Benner SA, Cohen MA, Gonnet GH. Amino acid substitution during functionally constrained divergent evolution of protein sequences. *Protein Engineering*. 1994;7(11):1323–1332. doi:10.1093/protein/7.11.1323. PMID: 7700864.
BLASTP	Biopython (substitution_matrices .load('BLASTP'))	blastp	Durbin R, Eddy SR, Krogh A, Mitchison G. *Biological Sequence Analysis: Probabilistic Models of Proteins and Nucleic Acids*. Cambridge University Press; 1998.
BLOSUM45	Biopython (substitution_matrices .load('BLOSUM45'))	blosum45	Henikoff S, Henikoff JG. Amino acid substitution matrices from protein blocks. *PNAS*. 1992;89(22):10915–10919. doi:10.1073/pnas.89.22.10915. PMID: 1438297.
BLOSUM50	Biopython (substitution_matrices .load('BLOSUM50'))	blosum50	Henikoff S, Henikoff JG. Amino acid substitution matrices from protein blocks. *PNAS*. 1992;89(22):10915–10919. doi:10.1073/pnas.89.22.10915. PMID: 1438297.
BLOSUM62	Biopython (substitution_matrices .load('BLOSUM62'))	blosum62	Henikoff S, Henikoff JG. Amino acid substitution matrices from protein blocks. *PNAS*. 1992;89(22):10915–10919. doi:10.1073/pnas.89.22.10915. PMID: 1438297.

BLOSUM80	Biopython (substitution_matrices .load('BLOSUM80'))	blosum80	Henikoff S, Henikoff JG. Amino acid substitution matrices from protein blocks. *PNAS*. 1992;89(22):10915–10919. doi:10.1073/pnas.89.22.10915. PMID: 1438297.
BLOSUM90	Biopython (substitution_matrices .load('BLOSUM90'))	blosum90	Henikoff S, Henikoff JG. Amino acid substitution matrices from protein blocks. *PNAS*. 1992;89(22):10915–10919. doi:10.1073/pnas.89.22.10915. PMID: 1438297.
DAYHOFF	Biopython (substitution_matrices .load('DAYHOFF'))	dayhoff	Dayhoff MO, Schwartz RM, Orcutt BC. A model of evolutionary change in proteins. *Atlas of Protein Sequence and Structure*, Vol. 5, Suppl. 3. 1978:345–352.
FENG	Biopython (substitution_matrices .load('FENG'))	feng	Feng DF, Johnson MS, Doolittle RF. Aligning amino acid sequences: comparison of commonly used methods. *Journal of Molecular Evolution*. 1984–1985;21(2):112–125. doi:10.1007/BF02100085. PMID: 6100188.
GENETIC	Biopython (substitution_matrices .load('GENETIC'))	genetic	Benner SA, Cohen MA, Gonnet GH. Amino acid substitution during functionally constrained divergent evolution of protein sequences. *Protein Engineering*. 1994;7(11):1323–1332. doi:10.1093/protein/7.11.1323. PMID: 7700864.
Gonnet1992	Biopython (substitution_matrices .load('Gonnet1992'))	gonnet1992	Gonnet GH, Cohen MA, Benner SA. Exhaustive matching of the entire protein sequence database. *Science*. 1992;256(5062):1443–1445. doi:10.1126/science.1604319. PMID: 1604319.
Grantham	Custom (https://gist.github.com/arq5x/5408712)	grantham	Grantham R. Amino acid difference formula to help explain protein evolution. *Science*. 1974;185(4154):862–864. doi:10.1126/science.185.4154.862. PMID: 4843792.
JOHNSON	Biopython (substitution_matrices .load('JOHNSON'))	johnson	Johnson MS, Overington JP. A structural basis for sequence comparisons. *Journal of Molecular Biology*. 1993;233(4):716–738. doi:10.1006/jmbi.1993.1548. PMID: 8411177.
JONES	Biopython (substitution_matrices .load('JONES'))	jones	Jones DT, Taylor WR, Thornton JM. The rapid generation of mutation data matrices from protein sequences. *Computational Applications in Biosciences*. 1992;8(3):275–282. doi:10.1093/bioinformatics/8.3.275. PMID: 1633570.

LEVIN	Biopython (substitution_matrices .load('LEVIN'))	levin	Levin JM, Robson B, Garnier J. An algorithm for secondary structure determination in proteins. *FEBS Letters*. 1986;205(2):303–308. doi:10.1016/0014-5793(86)80917-6. PMID: 3743779.
MCLACHLAN	Biopython (substitution_matrices .load('MCLACHLAN'))	mclachlan	McLachlan AD. Tests for comparing related amino-acid sequences. *Journal of Molecular Biology*. 1971;61(2):409–424. doi:10.1016/0022-2836(71)90390-1. PMID: 5167087.
MDM78	Biopython (substitution_matrices .load('MDM78'))	mdm78	Schwartz RM, Dayhoff MO. Matrices for detecting distant relationships. *Atlas of Protein Sequence and Structure*, Vol. 5, Suppl. 3. 1978:353–358.
PAM250	Biopython (substitution_matrices .load('PAM250'))	pam250	Dayhoff MO, Schwartz RM, Orcutt BC. A model of evolutionary change in proteins. *Atlas of Protein Sequence and Structure*, Vol. 5, Suppl. 3. 1978:345–352.
PAM30	Biopython (substitution_matrices .load('PAM30'))	pam30	Dayhoff MO, Schwartz RM, Orcutt BC. A model of evolutionary change in proteins. *Atlas of Protein Sequence and Structure*, Vol. 5, Suppl. 3. 1978:345–352.
PAM70	Biopython (substitution_matrices .load('PAM70'))	pam70	Dayhoff MO, Schwartz RM, Orcutt BC. A model of evolutionary change in proteins. *Atlas of Protein Sequence and Structure*, Vol. 5, Suppl. 3. 1978:345–352.
RAO	Biopython (substitution_matrices .load('RAO'))	rao	Mohana Rao JK. New scoring matrix for amino acid residue exchanges. *International Journal of Peptide and Protein Research*. 1987;29(2):276–281. doi:10.1111/j.1399-3011.1987.tb02254.x. PMID: 3570667.
RISLER	Biopython (substitution_matrices .load('RISLER'))	risler	Risler JL et al. Amino acid substitutions in structurally related proteins. *Journal of Molecular Biology*. 1988;204(4):1019–1029. doi:10.1016/0022-2836(88)90058-7. PMID: 3221397.
STR	Biopython (substitution_matrices .load('STR'))	str	Henikoff S, Henikoff JG. Performance evaluation of amino acid substitution matrices. *Proteins*. 1993;17(1):49–61. doi:10.1002/prot.340170108. PMID: 8234244.

Appendix Table S2. Performance comparison of different model architectures using the same input feature set. Models evaluated include LightGBM (used in VEFill), a fully connected neural network (FCNN), and a transformer-based architecture. Reported metrics include RMSE, MAE, and R^2 for both training and test sets. Variants of the FCNN included adjustments to regularization, architecture depth, and optimizer settings. All models were trained using the same 140-domain dataset.

Architecture / model	Train RMSE	Train MAE	Train R^2	Test RMSE	Test MAE	Test R^2
LightGBM (VEFill)	0.1399	0.1044	0.8349	0.2692	0.2040	0.4264
FCNN (reduced complexity, strong regularization)	0.1930	0.1443	0.6857	0.2666	0.1980	0.4375
FCNN (deeper, higher dropout, Adam optimizer)	0.2330	0.1772	0.5422	0.2726	0.2068	0.4117
Transformer (4 layers, 8 heads, d_model = 128)	0.1823	0.1338	0.7197	0.2723	0.2018	0.4130

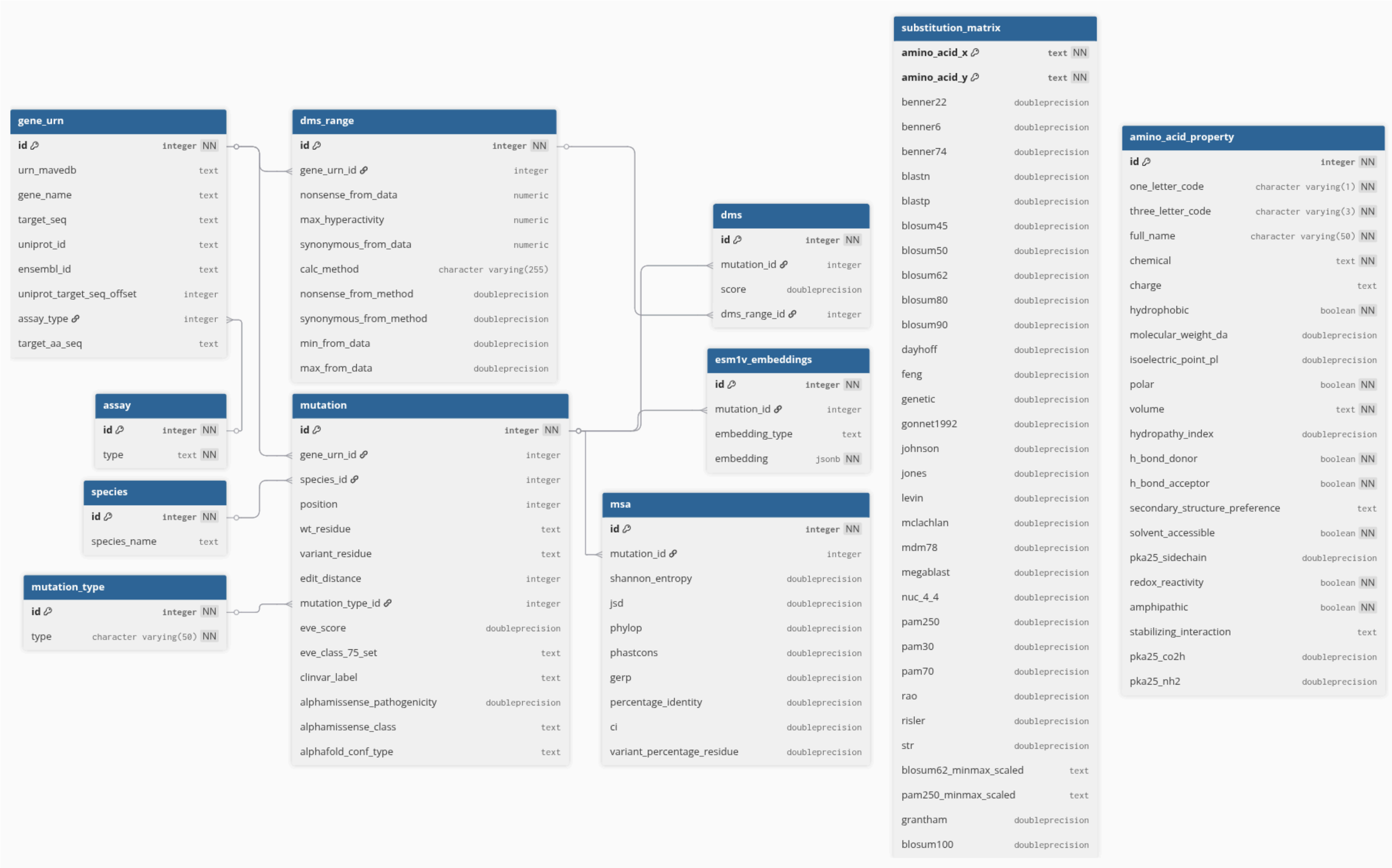

Appendix Figure S1. Schema of the custom PostgreSQL database developed for centralized storage and structured access to all model input features

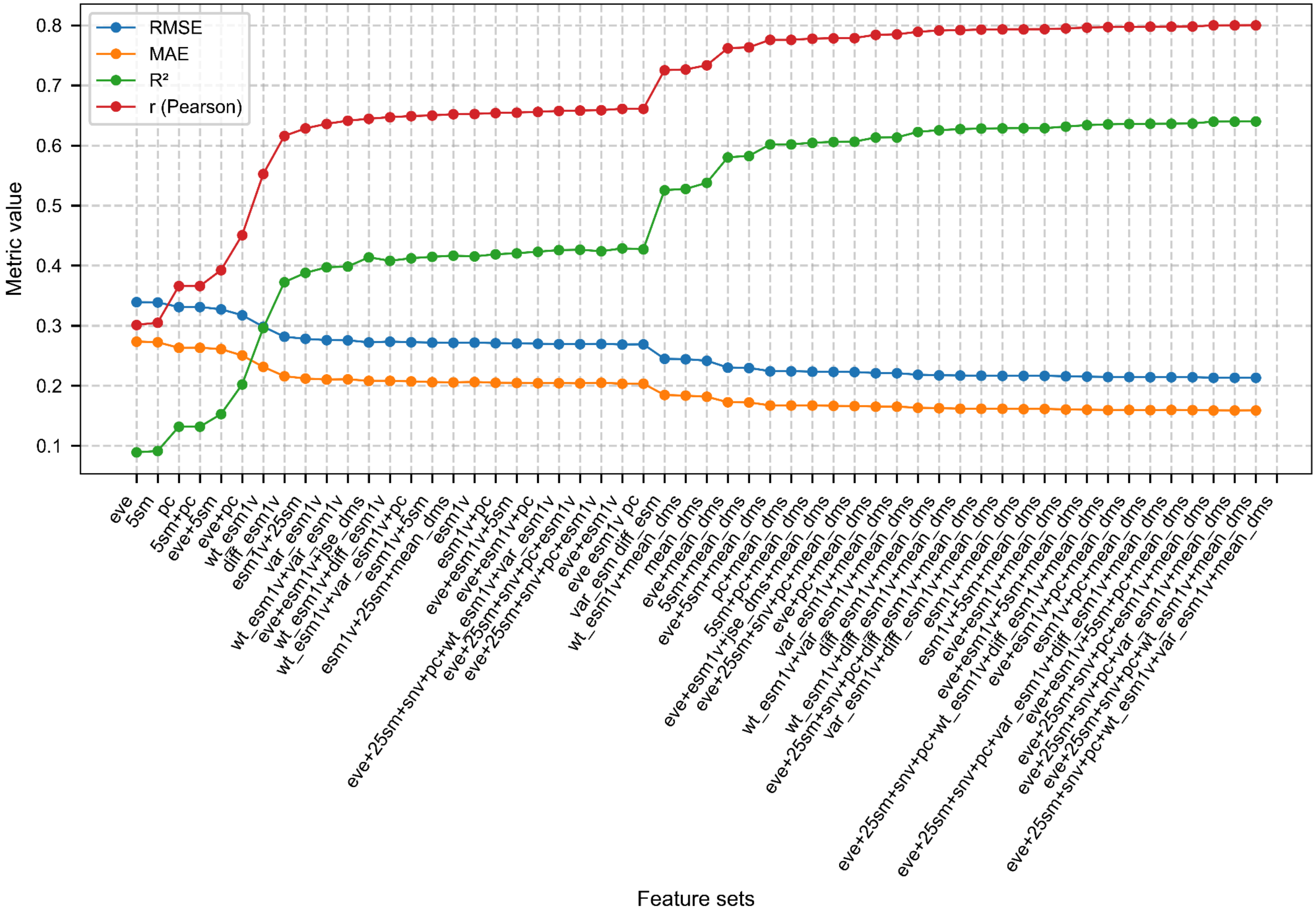

Appendix Figure S2. Performance metrics of the general cross-protein model trained and evaluated on various feature sets for DMS score imputation. Metrics shown include RMSE, MAE, R^2, and Pearson correlation coefficient (r). Feature sets on the x-axis are sorted by increasing Pearson correlation coefficient, highlighting the progressive improvements achieved by enriching the model with additional informative features.

Abbreviations used in the figure: esm: All ESM-1v embeddings (wild-type, variant, difference); wt_esm: ESM-1v embeddings for wild-type amino acid; var_esm: ESM-1v embeddings for variant amino acid; diff_esm: Difference between ESM-1v embeddings for wild-type and variant amino acid; mean_dms: Mean DMS score per amino acid position; jse_dms: James–Stein estimator of DMS score per position (global mean calculated per protein); pc: Physico-chemical properties (wild-type, variant, and their difference); sm: All substitution matrices (25 matrices); top5sm: Five substitution matrices (blosum62, blosum80, blosum90, grantham, gonnet1992); snv: Single-nucleotide variant indicator; eve: EVE scores.

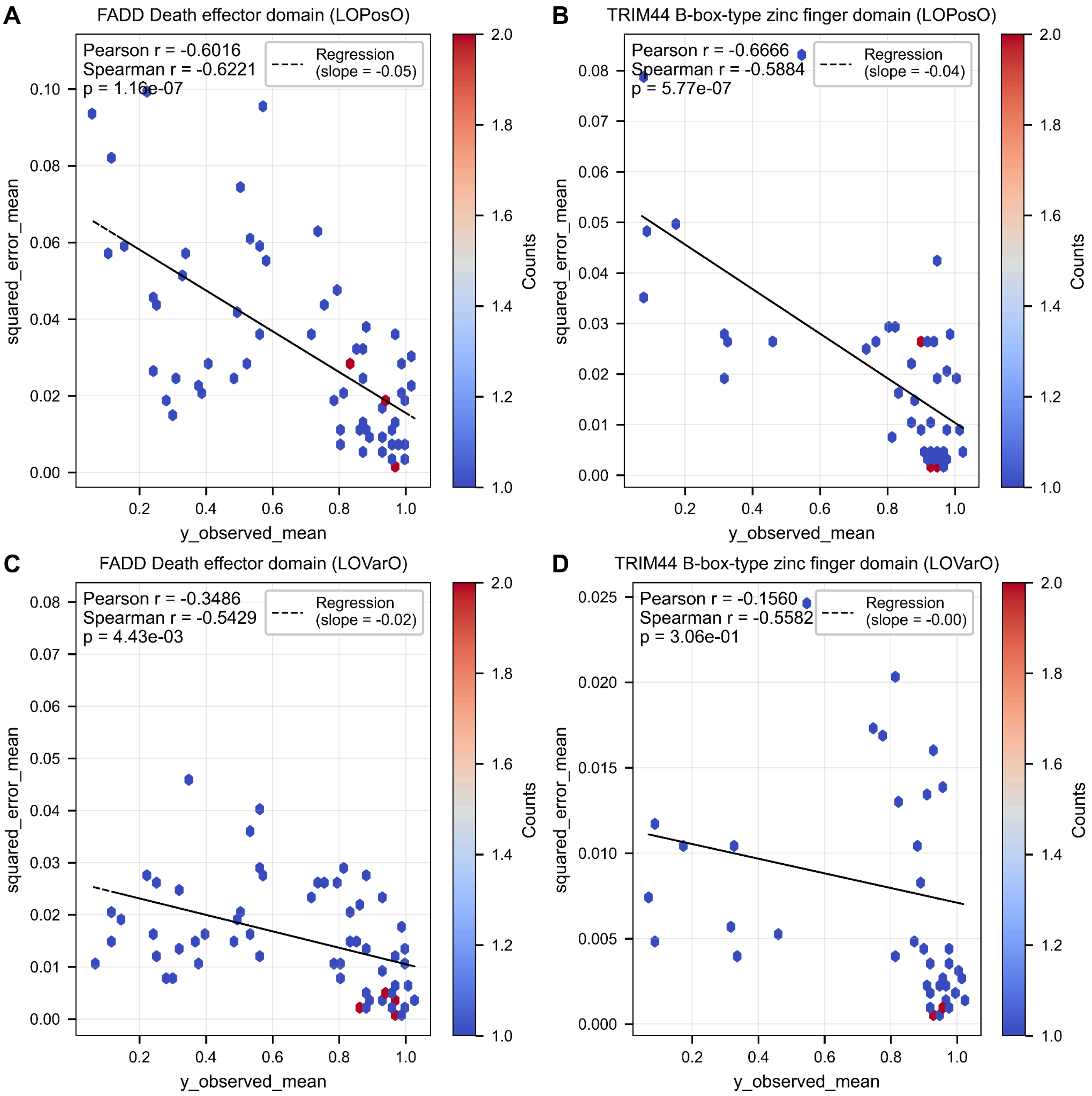

Appendix Figure S3. Correlation between average observed DMS scores and average prediction error across amino acid positions.

(A–B) Leave-one-position-out (LOPosO) model: Each point represents a single amino acid position. The x-axis shows the mean normalized DMS score (y_observed_mean) for all variants at that position, and the y-axis shows the mean squared error (squared_error_mean) of model predictions for those variants.

(C–D) Leave-one-variant-out (LOVarO) model: Each point again represents a single position, aggregating prediction errors across variants at that position where each variant was excluded once during training.

Strong negative correlations (Pearson r = −0.60 and −0.67) in LOPosO models for the FADD death effector domain **(A)** and the TRIM44 B-box-type zinc finger domain **(B)**, respectively, indicate that positions with lower (more damaging) DMS scores tend to have higher prediction errors. This suggests that LOPosO models struggle when predicting mutations at structurally or functionally critical positions due to the lack of positional context during training. Weaker negative correlations (Pearson r = −0.35 and −0.16) in LOVarO models for the same proteins, FADD **(C)** and TRIM44 **(D)**, demonstrate improved prediction accuracy when positional context is retained. The reduced error highlights the enhanced ability of these models to capture position-specific stability effects.

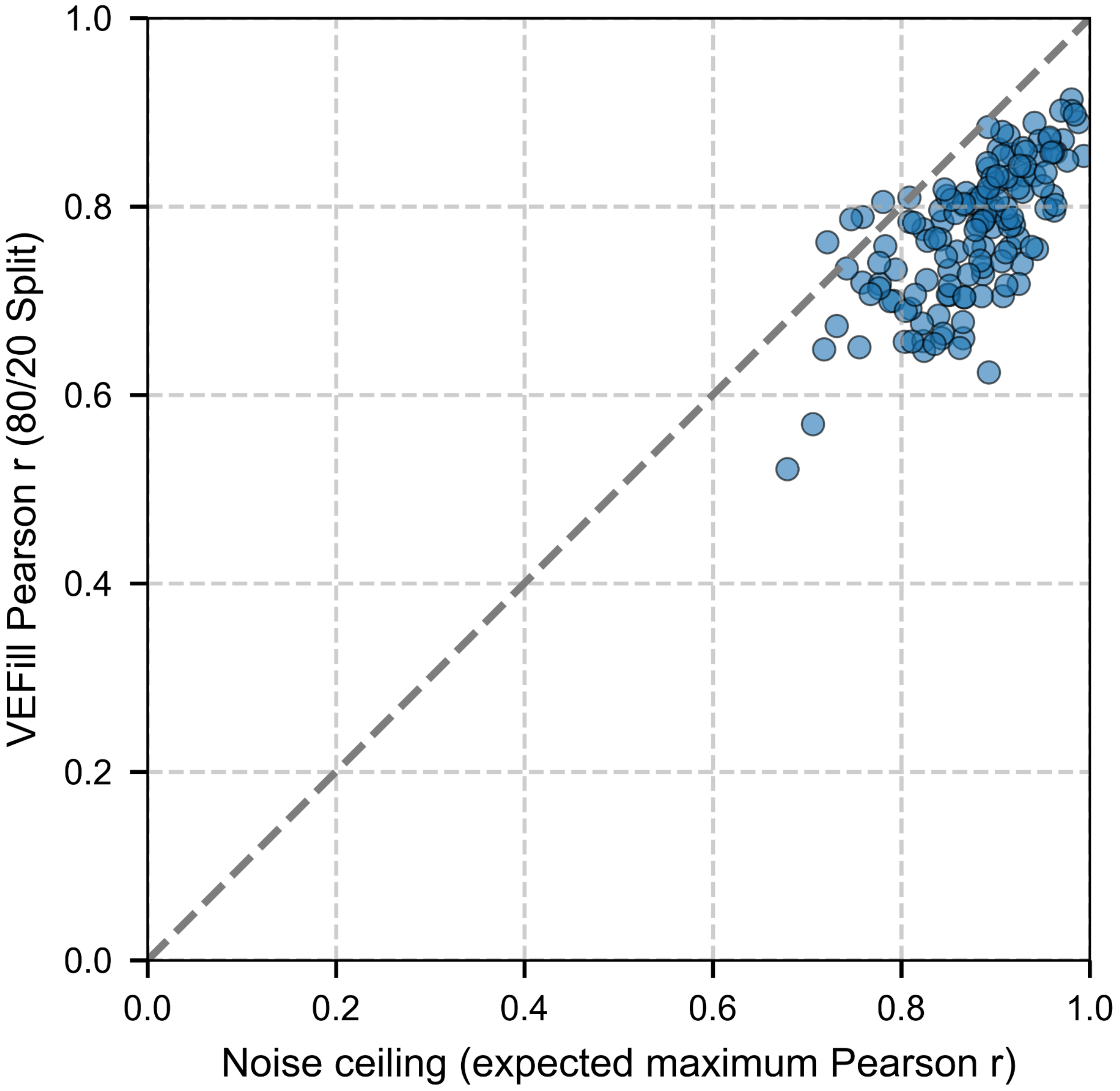

Appendix Figure S4. VEFill predictive accuracy versus the theoretical noise ceiling for 139 DMS datasets. Each point represents one protein, showing the VEFill Pearson correlation (80/20 split) plotted against the noise ceiling estimated via replicate-noise simulation (300 heteroscedastic Gaussian perturbations per dataset). The diagonal line represents the maximal achievable accuracy under the measured assay noise. Most proteins lie close to, but below, the noise ceiling, indicating that VEFill operates near the limit imposed by experimental uncertainty. Occasional points above the diagonal reflect expected sampling variability.

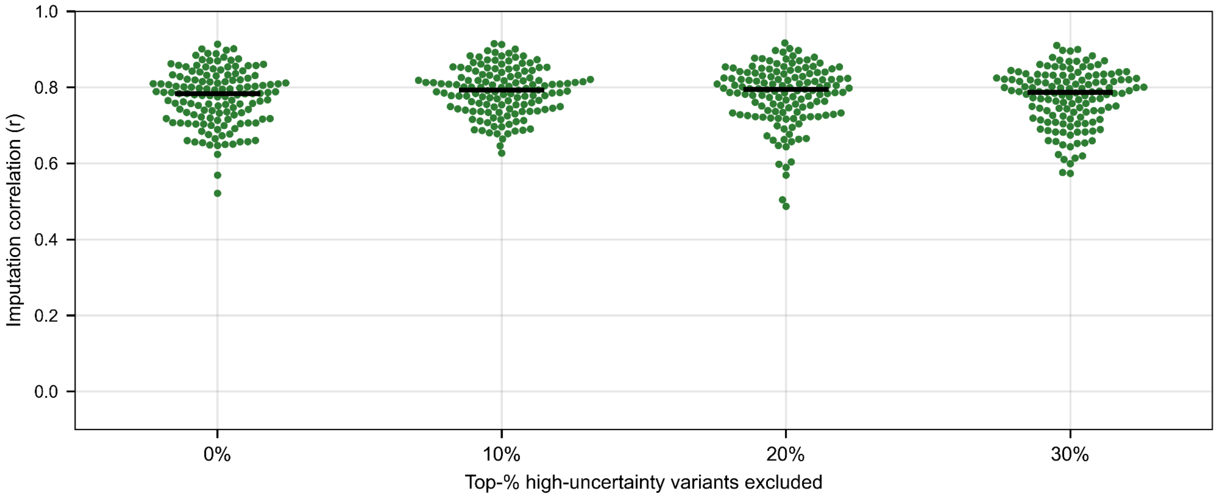

Appendix Figure S5. Effect of excluding high-uncertainty DMS variants from the 140-domain dataset on VEFill performance. Swarm plots show per-protein imputation accuracy (Pearson r) when excluding the top 0%, 10%, 20%, or 30% most uncertain variants, based on assay-reported measurement error. Black lines denote median correlations for each filtering level. Median performance remains stable across all conditions (r approximately 0.8), indicating that VEFill does not benefit from removing high-uncertainty measurements and is robust to experimental noise in the input data.

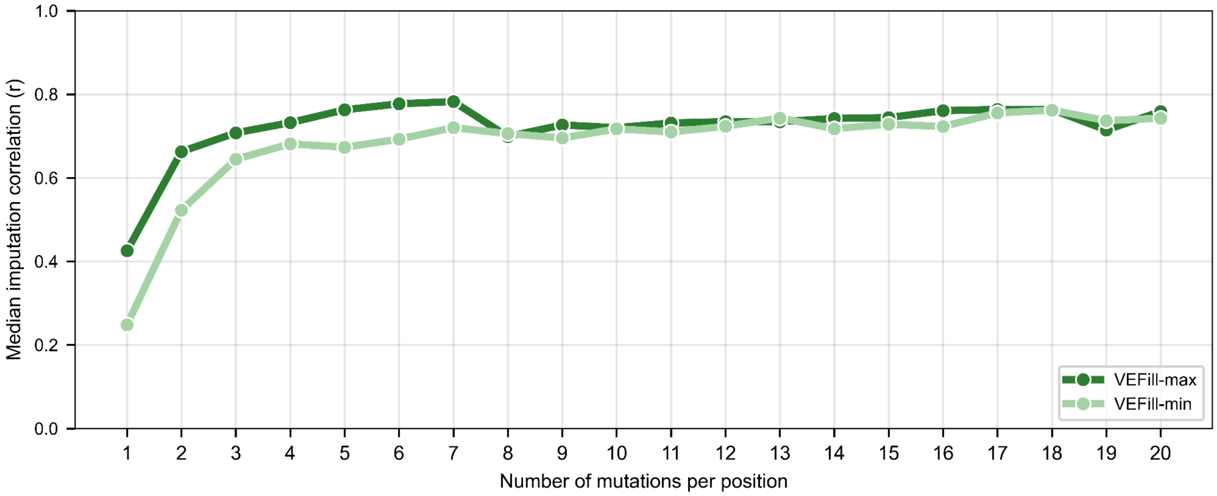

Appendix Figure S6. Effect of the number of mutations per position on VEFill performance. Per-protein imputation accuracy as a function of the number of experimentally measured substitutions available per position. VEFill-min and VEFill-max were trained on 28 high-quality DMS datasets while restricting training data to a fixed number of mutations per position (N = 1–20), randomly sampled for each site. Positional mean DMS scores were recomputed dynamically using only the available mutations at each position. Plotted values represent the median Pearson correlation between predicted and experimental DMS scores across all datasets.

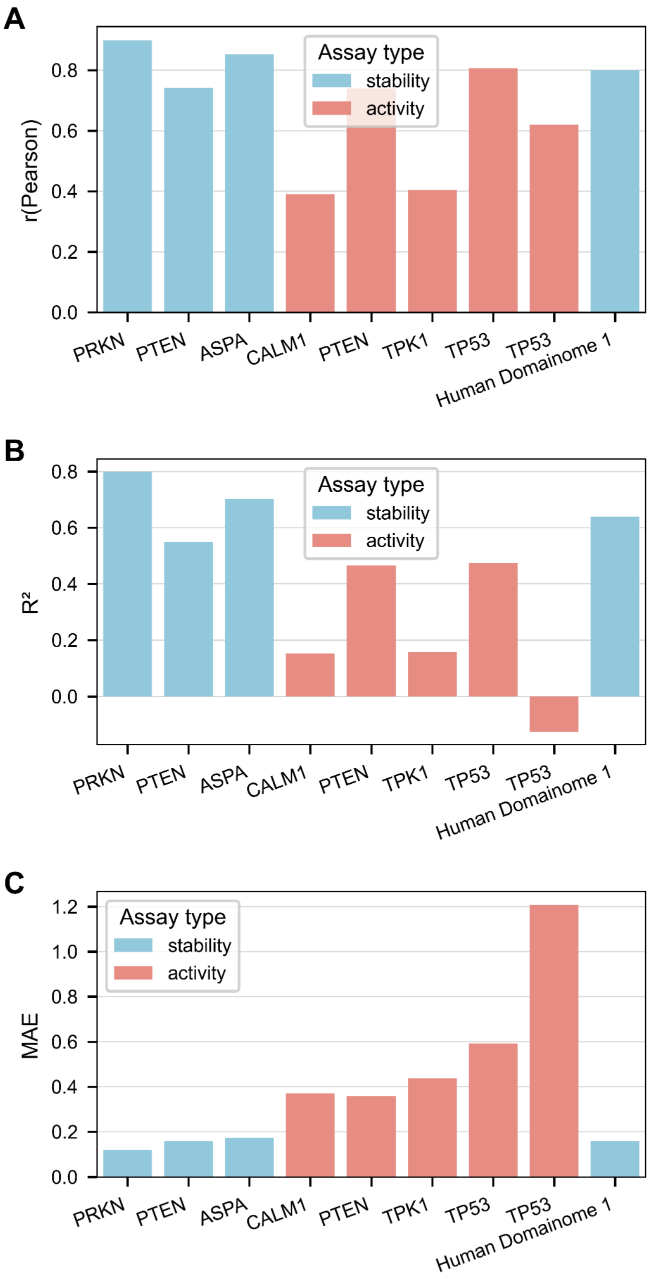

Appendix Figure S7. Comparison of predictive performance metrics of the general cross-protein model (trained on the Human Domainome 1 dataset) across external stability-based assays and activity-based assays. (A) Pearson correlation (r), **(B)** R^2, and **(C)** MAE shown individually for each assay.

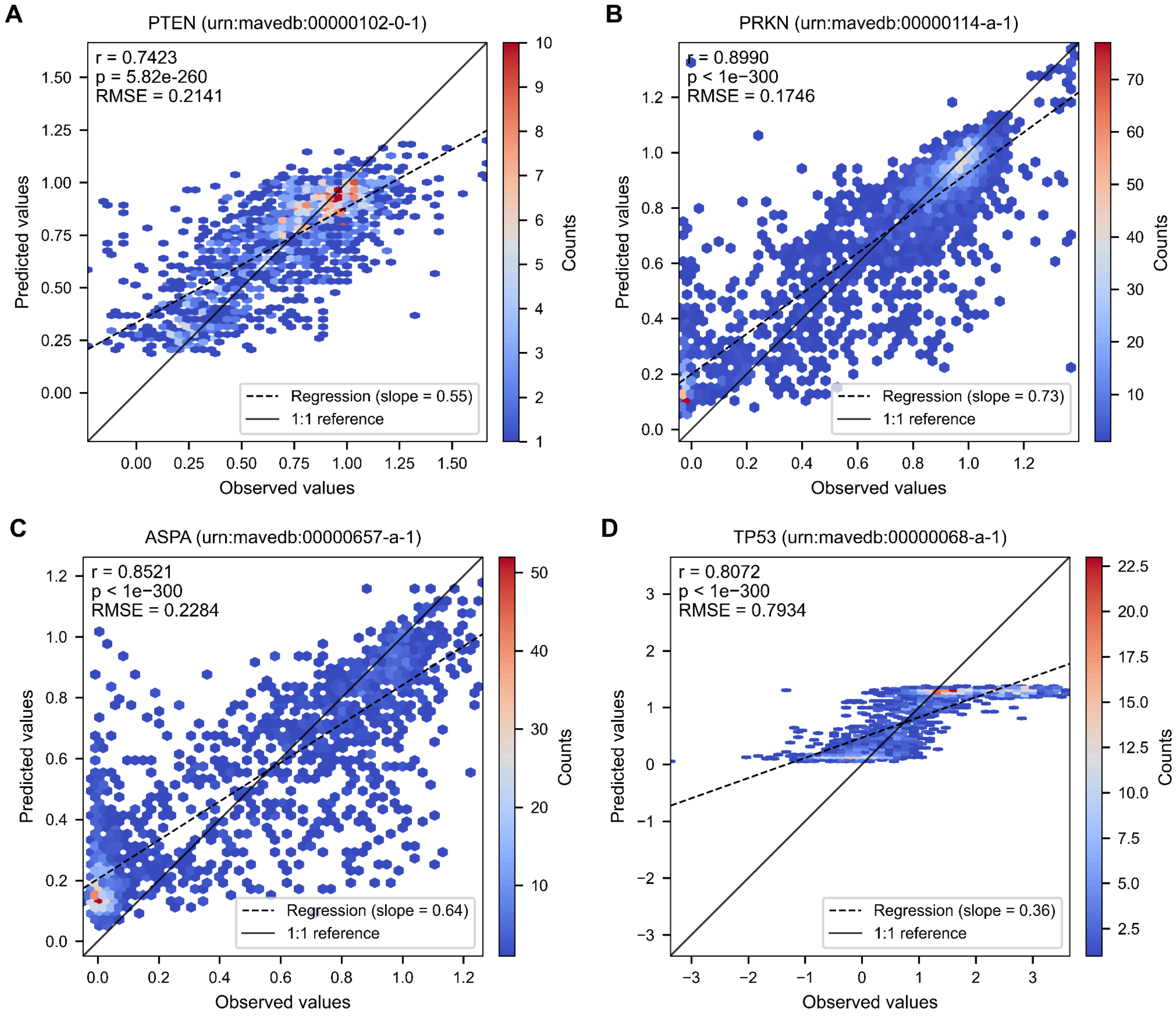
A
PTEN (urn:mavedb:00000102-0-1)
r = 0.7423
p = 5.82e-260
RMSE = 0.2141
Regression (slope = 0.55)
1:1 reference
Predicted values
Observed values
Counts
B
PRKN (urn:mavedb:00000114-a-1)
r = 0.8990
p < 1e−300
RMSE = 0.1746
Regression (slope = 0.73)
1:1 reference
C
ASPA (urn:mavedb:00000657-a-1)
r = 0.8521
p < 1e−300
RMSE = 0.2284
Regression (slope = 0.64)
1:1 reference
D
TP53 (urn:mavedb:00000068-a-1)
r = 0.8072
p < 1e−300
RMSE = 0.7934
Regression (slope = 0.36)
1:1 reference

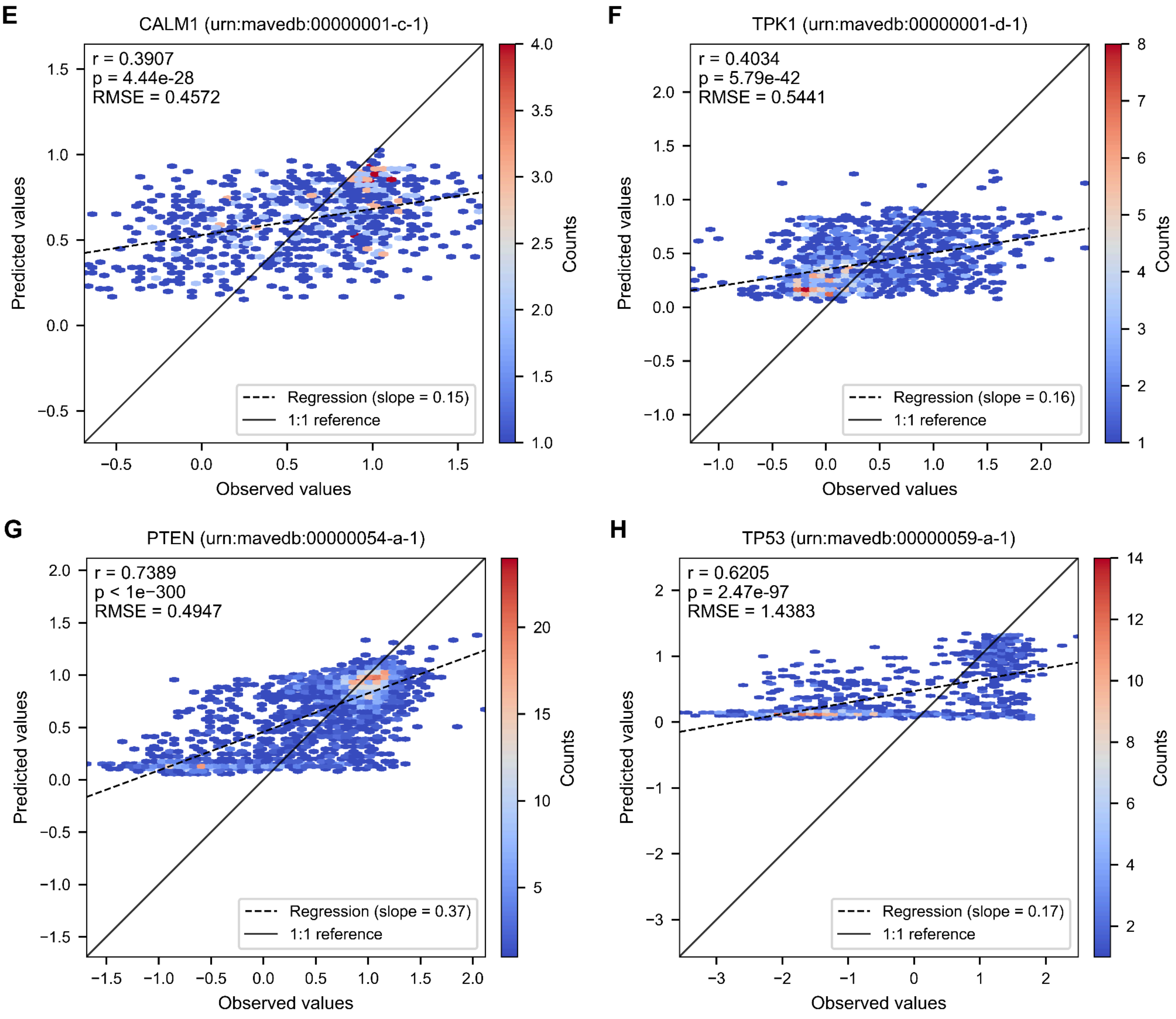

Appendix Figure S8. Comparison of predicted and experimentally measured normalized DMS scores for the general cross-protein model trained on the Human Domainome 1 dataset, tested on external stability-based assays (A–C) and activity-based assays (D–H). Each plot represents the density of observations by color intensity, with the 1:1 reference line indicating perfect predictions (y = x). Stability-based assays **(A–C)** show strong predictive accuracy with high correlations (Pearson $r \geq 0.74$) and low RMSE: **(A)** PTEN, **(B)** PRKN, **(C)** ASPA. Activity-based assays **(D–H)** generally exhibit weaker predictive performance with higher RMSE and variable correlation: **(D)** TP53, **(E)** CALM1, **(F)** TPK1, **(G)** PTEN activity-based assay, and (H) TP53. Poor performance is especially evident for gain-of-function mutations.

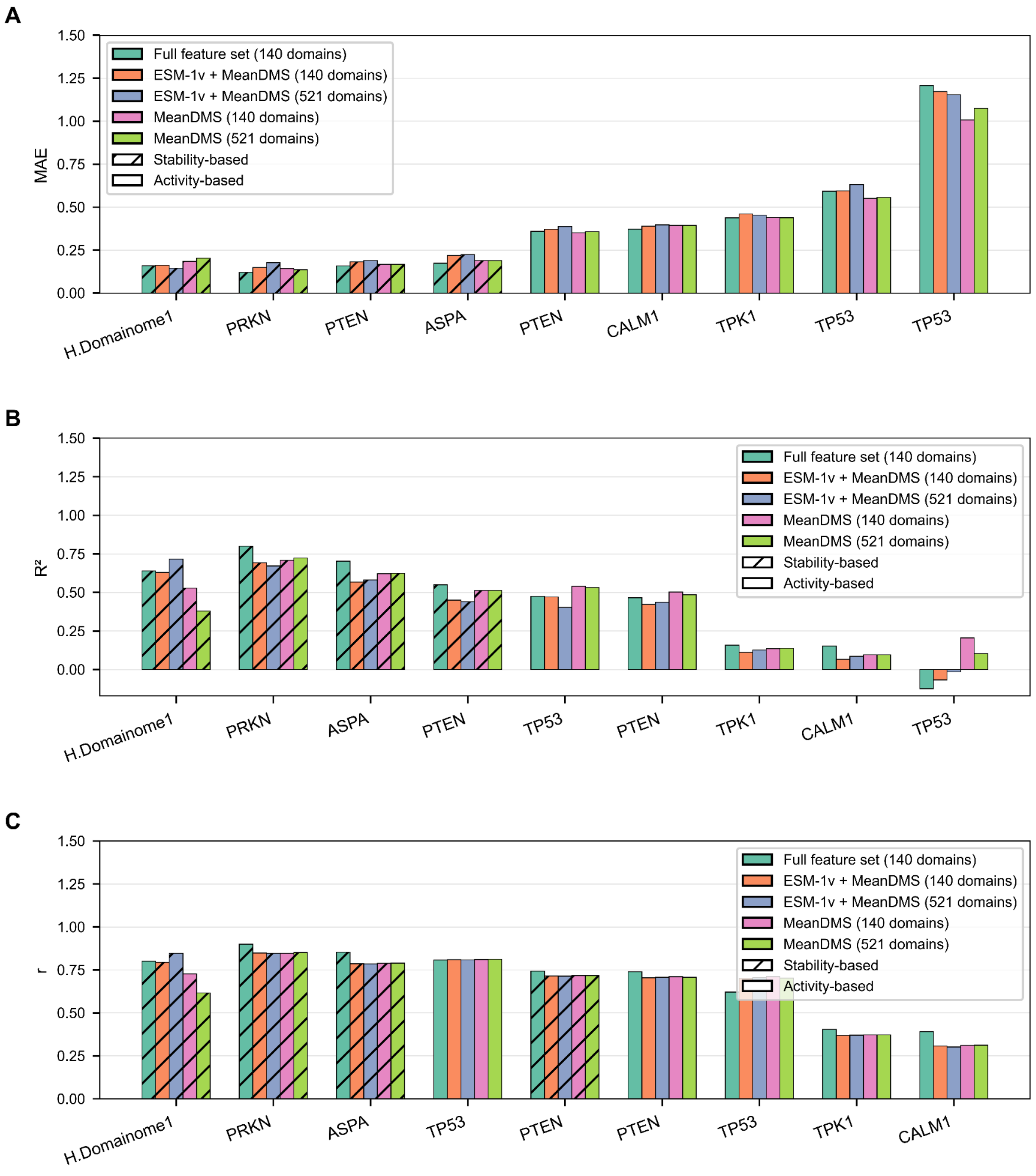

Appendix Figure S9. Performance of full-feature versus reduced-feature set models trained on 140 and 521 domains, evaluated on Human Domainome 1 and unseen full-length proteins. Subfigures **(A–C)** present evaluation metrics comparing model performance on held-out Human Domainome 1 domains and full-length non-Domainome proteins (PRKN, PTEN, ASPA, CALM1, TPK1, and TP53). Bars show results for the full-feature model, the ESM-1v plus mean DMS model, and the mean DMS-only model. Stability-based assays are indicated by patterned bar fills. **(A)** MAE, **(B)** Coefficient of determination (R^2), **(C)** Pearson correlation coefficient r.

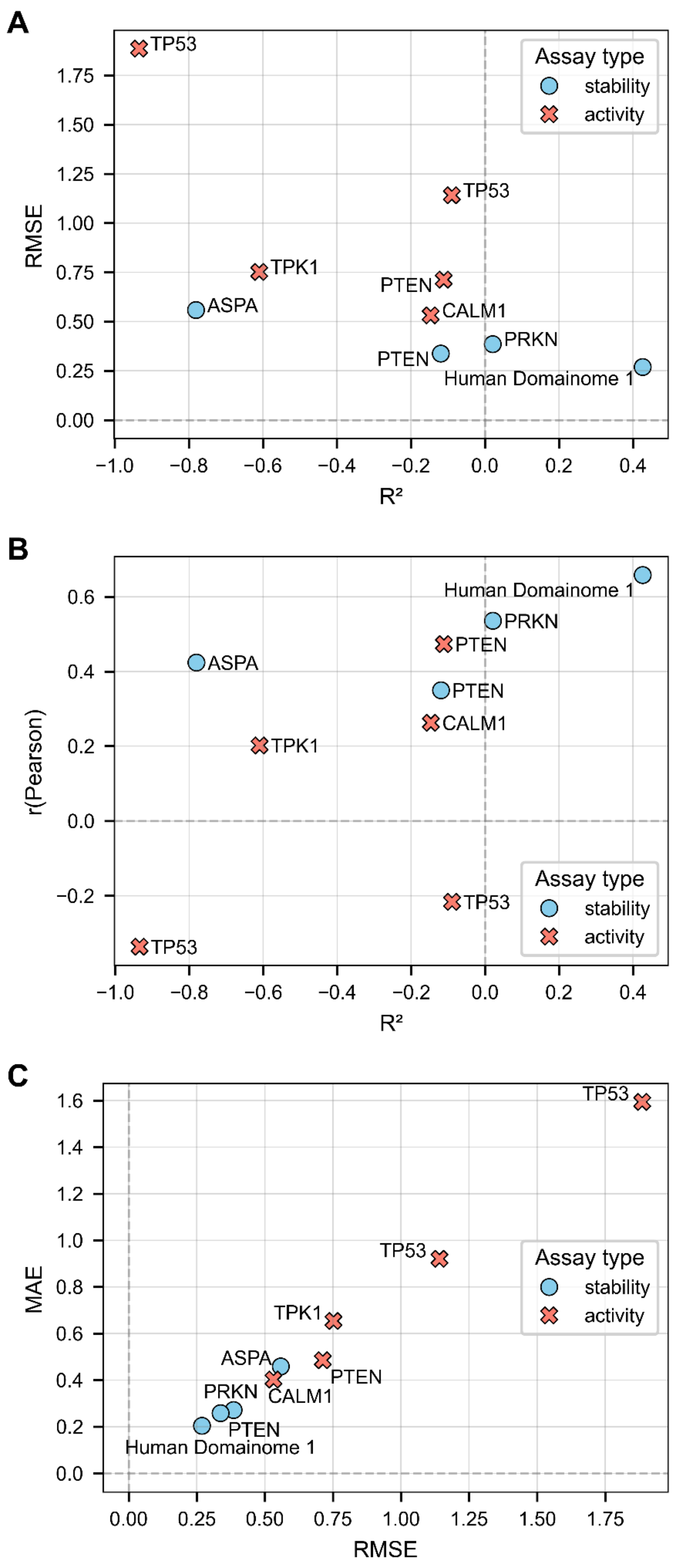

Appendix Figure S10. Performance metrics for the zero-shot general cross-protein model trained without positional mean DMS scores, evaluated on external stability-based and activity-based assays. (A) RMSE versus R^2. **(B)** Pearson correlation (r) versus R^2. **(C)** MAE versus RMSE. Across all metrics, stability-based assays show higher predictive accuracy than activity-based assays. Internal validation on the Domainome dataset is provided as a baseline reference.

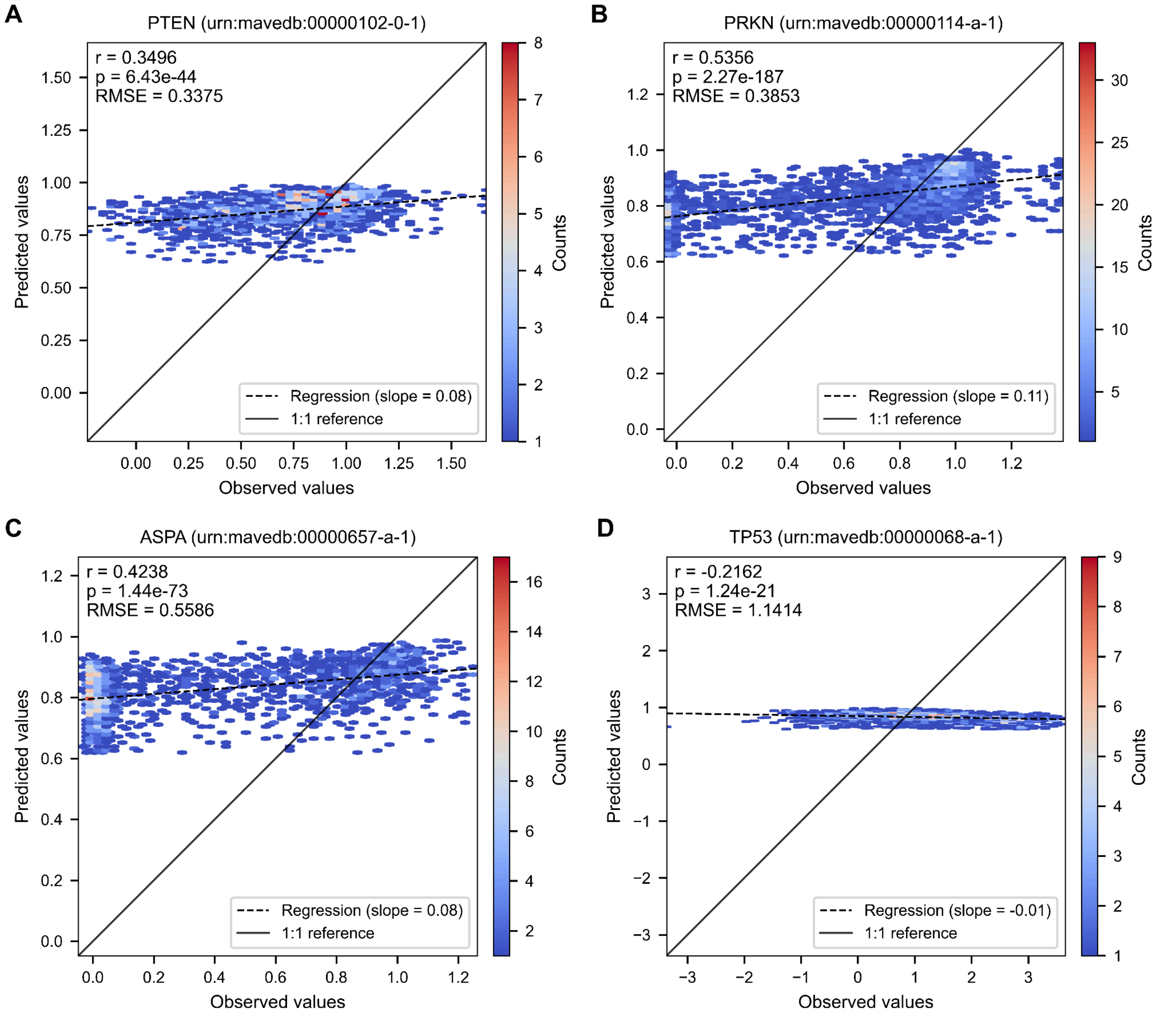
A
PTEN (urn:mavedb:00000102-0-1)
r = 0.3496
p = 6.43e-44
RMSE = 0.3375
Predicted values
Observed values
Counts
Regression (slope = 0.08)
1:1 reference
B
PRKN (urn:mavedb:00000114-a-1)
r = 0.5356
p = 2.27e-187
RMSE = 0.3853
Predicted values
Observed values
Counts
Regression (slope = 0.11)
1:1 reference
C
ASPA (urn:mavedb:00000657-a-1)
r = 0.4238
p = 1.44e-73
RMSE = 0.5586
Predicted values
Observed values
Counts
Regression (slope = 0.08)
1:1 reference
D
TP53 (urn:mavedb:00000068-a-1)
r = -0.2162
p = 1.24e-21
RMSE = 1.1414
Predicted values
Observed values
Counts
Regression (slope = -0.01)
1:1 reference

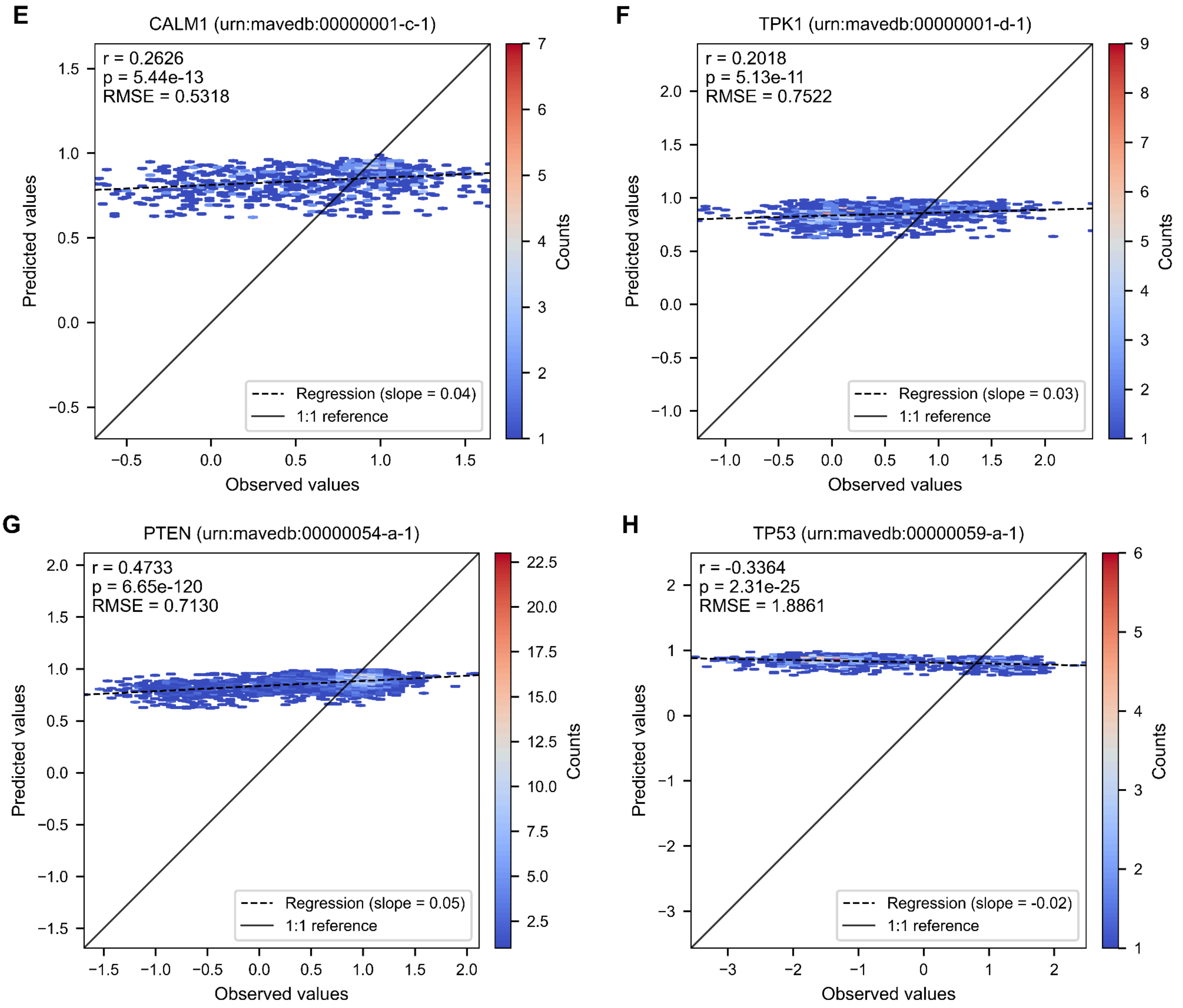

Appendix Figure S11. Zero-shot predictions (without positional mean DMS scores) versus experimentally measured normalized DMS scores for external assays. Color intensity indicates data density, and dashed lines represent perfect predictions (y = x). Stability-based assays **(A–C)**: **(A)** PTEN, **(B)** PRKN, **(C)** ASPA show moderate correlations (Pearson r approximately 0.34–0.54), but reduced accuracy compared to the full-feature general cross-protein model. Activity-based assays **(D–H)**: **(D)** TP53, **(E)** CALM1, **(F)** TPK1, **(G)** PTEN functional assay, and **(H)** TP53 show substantially weaker performance, higher prediction error, and very low or negative correlations.
