# Supplementary material for: VEFill: accurate and generalizable deep mutational scanning score imputation across protein domains
Source: Mol Syst Biol. 2026 Mar 20;22(6):979–1002. doi: 10.1038/s44320-026-00203-y (PMC13230771; doi:10.1038/s44320-026-00203-y)
Supplement: Supplementary file 22 — Expanded View Figures [file 44320_2026_203_MOESM22_ESM.pdf]

Expanded View Figures

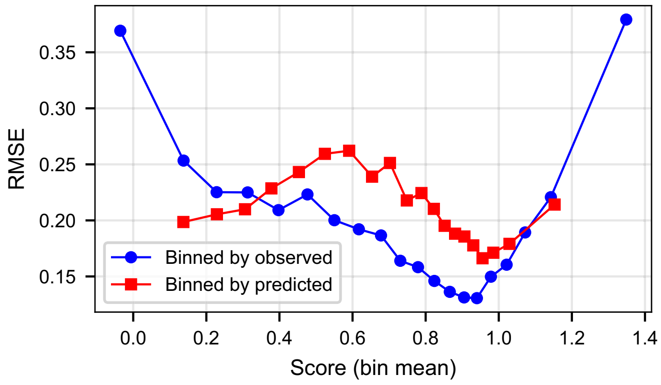

Figure EV1. RMSE computed in quantile-based bins using either observed or predicted DMS scores.

The observed-binned curve reveals variation in predictive accuracy across the biological effect spectrum, while the predicted-binned curve reflects model calibration across its output range Source data are available online for this figure.

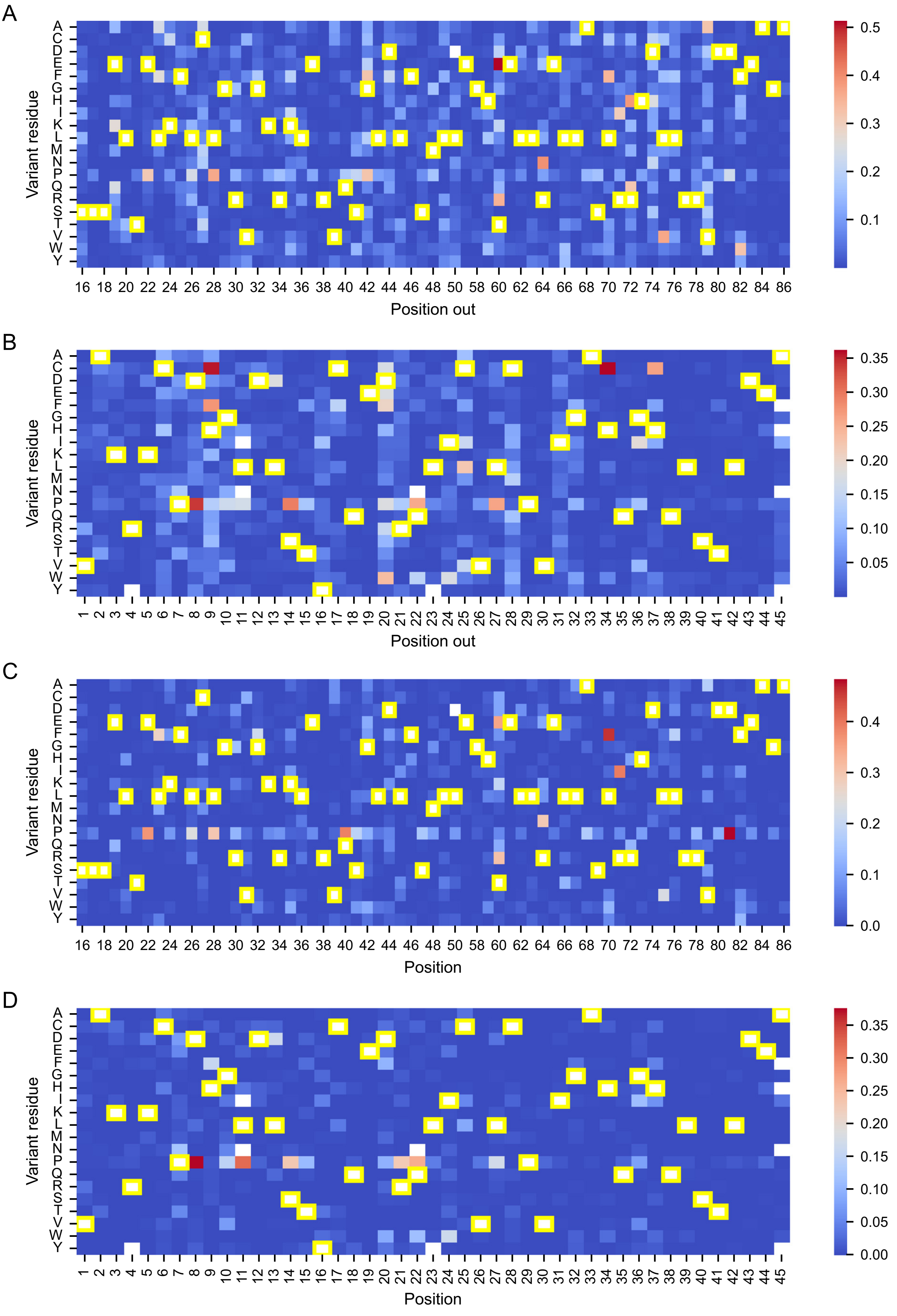
A
Variant residue
A C D E F G H I K L M N P Q R S T V W Y
16 18 20 22 24 26 28 30 32 34 36 38 40 42 44 46 48 50 58 60 62 64 66 68 70 72 74 76 78 80 82 84 86
Position out
0.5 0.4 0.3 0.2 0.1
B
Variant residue
A C D E F G H I K L M N P Q R S T V W Y
1 2 3 4 5 6 7 8 9 10 11 12 13 14 15 16 17 18 19 20 21 22 23 24 25 26 27 28 29 30 31 32 33 34 35 36 37 38 39 40 41 42 43 44 45
Position out
0.35 0.30 0.25 0.20 0.15 0.10 0.05
C
Variant residue
A C D E F G H I K L M N P Q R S T V W Y
16 18 20 22 24 26 28 30 32 34 36 38 40 42 44 46 48 50 58 60 62 64 66 68 70 72 74 76 78 80 82 84 86
Position
0.4 0.3 0.2 0.1 0.0
D
Variant residue
A C D E F G H I K L M N P Q R S T V W Y
1 2 3 4 5 6 7 8 9 10 11 12 13 14 15 16 17 18 19 20 21 22 23 24 25 26 27 28 29 30 31 32 33 34 35 36 37 38 39 40 41 42 43 44 45
Position
0.35 0.30 0.25 0.20 0.15 0.10 0.05 0.00

Figure EV2. The squared error between predicted and observed normalized DMS scores for the two best-performing per-protein models under LOPosO and LOVarO strategies.

(**A**, **B**) SE distributions for FADD and TRIM44, respectively, under the LOPosO strategy, where in each iteration, all variants at a single position are held out for testing. The squared error for each variant at the held-out position is calculated and plotted in the heatmap. This process is repeated across all positions, holding out a different position in each iteration. (**C**, **D**) SE distributions for FADD and TRIM44, respectively, using the LOVarO strategy, in which only one variant is held out at a time. The SE is calculated for that single held-out variant and plotted accordingly. This fine-grained approach evaluates the model's ability to predict individual unseen mutations. Cells with yellow frames indicate the wild-type amino acid at each position Source data are available online for this figure.

© The Author(s)

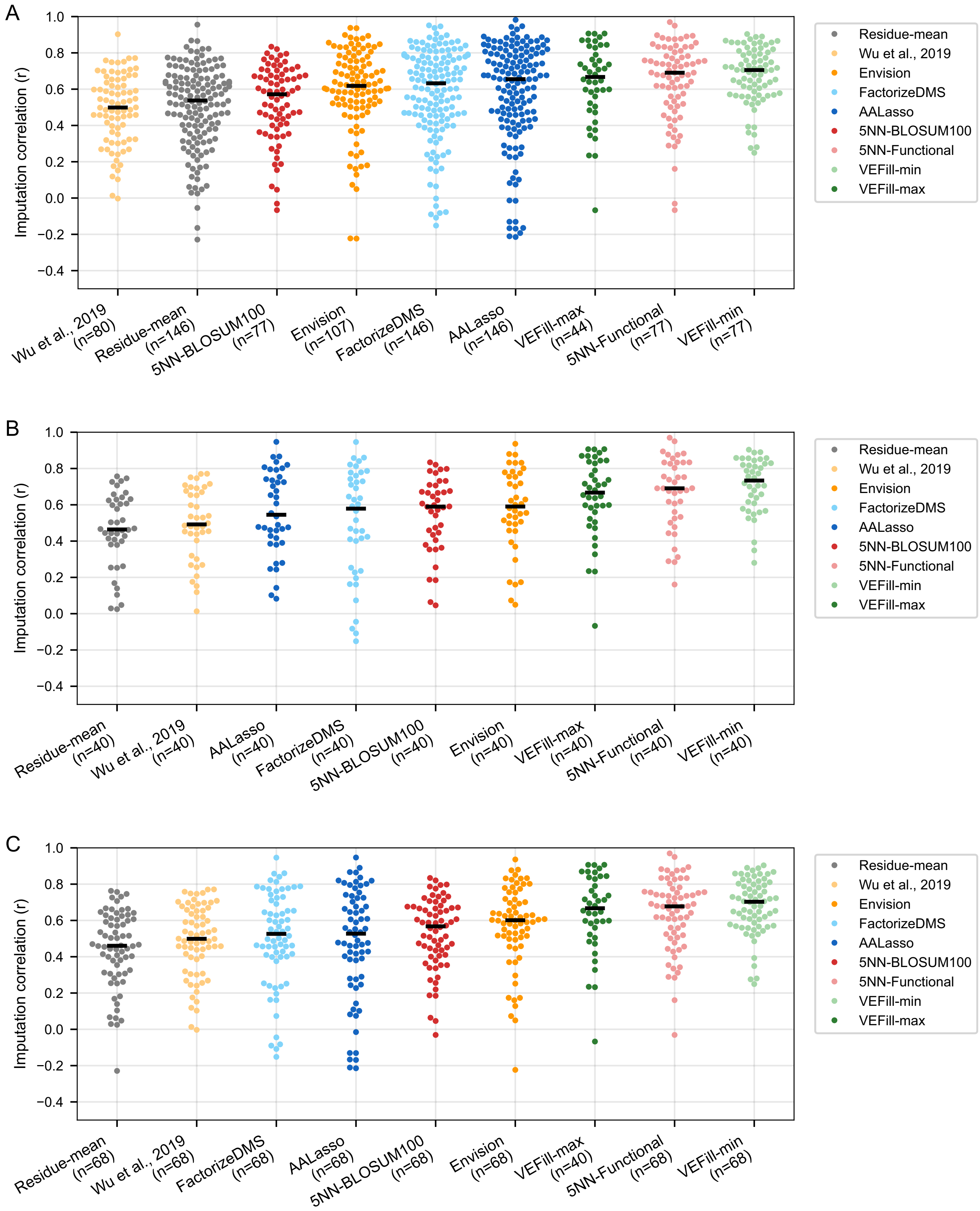
A
B
C
Imputation correlation (r)
1.0
0.8
0.6
0.4
0.2
0.0
−0.2
−0.4
Wu et al., 2019 (n=80)
Residue-mean (n=146)
5NN-BLOSUM100 (n=77)
Envision (n=107)
FactorizeDMS (n=146)
AALasso (n=146)
VEFill-max (n=44)
5NN-Functional (n=77)
VEFill-min (n=77)
Residue-mean (n=40)
Wu et al., 2019 (n=40)
AALasso (n=40)
FactorizeDMS (n=40)
5NN-BLOSUM100 (n=40)
Envision (n=40)
VEFill-max (n=40)
5NN-Functional (n=40)
VEFill-min (n=40)
Residue-mean (n=68)
Wu et al., 2019 (n=68)
FactorizeDMS (n=68)
AALasso (n=68)
5NN-BLOSUM100 (n=68)
Envision (n=68)
VEFill-max (n=40)
5NN-Functional (n=68)
VEFill-min (n=68)
Residue-mean
Wu et al., 2019
Envision
FactorizeDMS
AALasso
5NN-BLOSUM100
5NN-Functional
VEFill-min
VEFill-max

 © The Author(s)

Figure EV3. Benchmarking of imputation models across DMS datasets.

(**A**) All 146 proteins with available features were included in the analysis. Because some models require specific annotations (e.g., structural or evolutionary features), not all models could be trained on all proteins; each method is therefore evaluated on the maximal subset of datasets for which its requisite features were available. (**B**) This analysis includes only the 40 proteins for which all benchmarking models had complete feature availability. Each model was trained and evaluated on the exact same set of proteins, enabling strict, like-for-like comparison without confounding differences in dataset coverage. (**C**) This panel includes the 68 proteins for which all benchmarking models had complete feature coverage. Because VEFill-max requires EVE scores, which were unavailable for a subset of proteins, it could only be trained and evaluated on the 40 proteins (as shown in (**B**)), whereas all other models were evaluated on the full 68-protein set. A simple Residue-mean baseline—imputing a variant's effect as the mean DMS score of all other variants at the same position—is included for reference. Each point in the swarm plot represents the per-protein Pearson correlation between predicted and experimental DMS scores for that model. Differences in point counts across models reflect differences in feature availability across datasets Source data are available online for this figure.

© The Author(s)

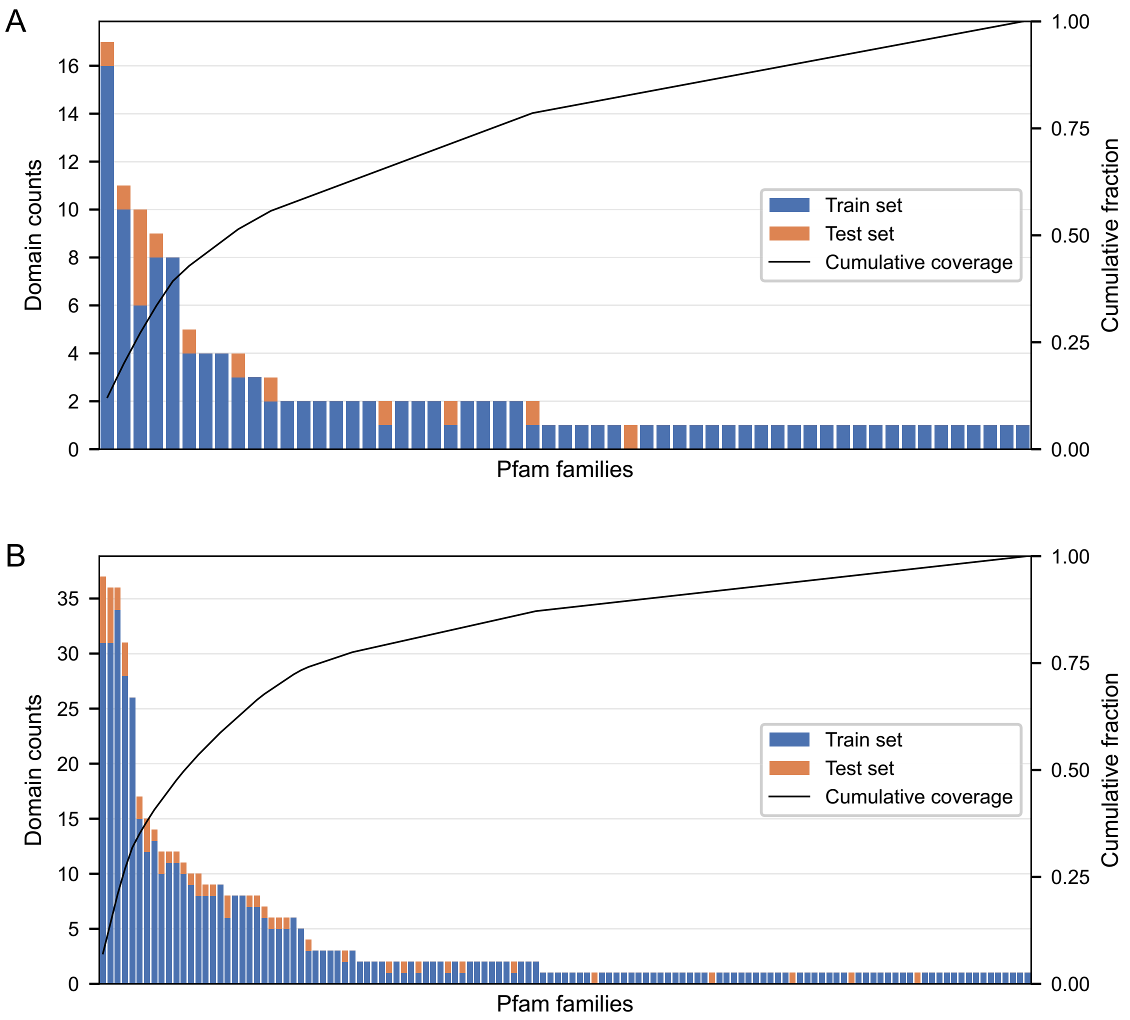

Figure EV4. Distribution of protein families in the 140-domain and 521-domain training datasets used for reduced-feature model training.

(**A**) 140-domain dataset; (**B**) 521-domain dataset. The left Y-axis indicates the number of protein domains per protein family. Bars are stacked to show counts in the training and test sets. Pfam IDs are ordered by descending total domain count. The right Y-axis shows the cumulative fraction of the dataset accounted for by each Pfam family, highlighting the dominance of a small subset of Pfam families. (See Dataset EV1 for detailed list of domains, including UniProt IDs, Pfam IDs, and their presence in the 140- and 521-domain datasets) Source data are available online for this figure.

 © The Author(s)
